# Simultaneous measurement of tadehaginoside and its principal metabolite in rats by HPLC–MS/MS and its application in pharmacokinetics and tissue distribution study

**DOI:** 10.1080/13880209.2021.1990354

**Published:** 2021-10-24

**Authors:** Cai-Yun Zhang, Ya-Ting Lu, Yin-Feng Tan, Qi-Bing Liu, Lin Dong, Ning Ma, Wei-Ying Lu, Zhi-Heng Su, Xiao-Po Zhang

**Affiliations:** a Key Laboratory of Tropical Translational Medicine of Ministry of Education, Hainan Key Laboratory for Research and Development of Tropical Herbs, School of Pharmacy, Hainan Medical University, Haikou, P. R. China; b Pharmaceutical College, Guangxi Medical University, Nanning, China; c The First People’s Hospital of Nanning, Nanning, China; d Reproductive Medical Center, Hainan Women and Children’s Medical Center, Haikou, China

**Keywords:** *p*-Hydroxycinnamic, method development and validation, metabolic profile

## Abstract

**Context:**

Tadehaginoside, an active ingredient isolated from *Tadehagi triquetrum* (Linn.) Ohashi (Leguminosae), exhibited various biological activities. However, the pharmacokinetics and tissue distribution which affect tadehaginoside’s therapeutic actions and application remain elusive.

**Objective:**

To clarify the metabolism of tadehaginoside *in vivo*.

**Materials and methods:**

The pharmacokinetics and tissue distribution of tadehaginoside and its metabolite *p*-hydroxycinnamic acid (HYD) were investigated using LC-MS/MS. Pharmacokinetic parameters were determined in 10 Sprague-Dawley rats divided into two groups, the intravenous group (5 mg/kg) and the oral group (25 mg/kg). For the tissue-distribution study, 20 rats were intravenously given tadehaginoside (5 mg/kg) before the experiment (*n* = 4). Biological samples were collected before drug administration (control group) and after drug administration.

**Results:**

The linearity, accuracy, precision, stability, recovery and matrix effect of the method were well-validated and the results satisfied the requirements of biological sample measurement. Treatment with tadehaginoside *via* intragastric and intravenous administration, the calculated *C*_max_ in rats was 6.01 ± 2.14 ng/mL and 109.77 ± 4.29 ng/mL, and *T*_max_ was 0.025 ± 0.08 h and 0.08 h, respectively. The results indicated that the quick absorption of tadehaginoside was observed following intravenous administration, and tadehaginoside in plasma of rats with intragastric administration showed relatively low concentration may be due to the formation of its metabolite. Tissue-distribution study indicated that kidney and spleen were the major distribution organs for tadehaginoside in rats and there was no long-term accumulation in most tissues.

**Discussion and conclusion:**

These results could provide clues for exploring the bioactivity of tadehaginoside based on its pharmacokinetic characteristics.

## Introduction

*Tadehagi triquetrum* (Linn.) Ohashi (Leguminosae), as a traditional Chinese medicine in the southwest of China, has been used extensively in treatment for cold and fever, sore throat, lung sputum, jaundice, hepatitis, nephritis, and enteritis (Wu et al. 2015; Aye et al. 2019; Wang et al. 2019). Tadehaginoside was obtained from this plant and assigned as its main active constituent, which comprises a glucosyl, phloroglucinol, and a *trans-p*-hydroxycinnamoyl moiety. Recent studies have revealed that tadehaginoside displayed a broad spectrum of biological activities and was deemed as a lead compound for further drug development. For example, it has been demonstrated that tadehaginoside could reduce CCl_4_-induced oxidative damage and inflammatory through the Nrf2 signalling pathway and NF-κB pathway (Tang et al. 2014). In addition, tadehaginoside displayed the ability in increasing glucose uptake by up-regulating PPARγ and glucose transporter-4 in C2C12 Myotubes (Zhang et al. 2016). Besides, tadehaginoside exhibited biological activities in decreasing the accumulation of lipid in HepG2 cells (Zhang et al. 2015). Moreover, tadehaginoside showed potent activities in curing metabolic disease and could be used as a therapeutic agent against obesity, diabetes, and atherosclerosis (Maison et al. 2014; Zhang et al. 2015).

Our previous study partially explored the identification of tadehaginoside and its metabolites, and preliminarily determined that *p*-hydroxycinnamic acid (HYD) was the major metabolite of tadehaginoside in rats (Zhang et al. 2015). It is reported that HYD also showed strong antidiabetic and antihyperlipidemic effects by modulating the level of glucose, triglyceride, and total cholesterol (Amalan et al. 2016; Zabad et al. 2019). Tadehaginoside and its metabolite HYD are similar in pharmacological activities, but it is still unclear that which one plays the major role *in vivo* after treatment with tadehaginoside. Due to the novel chemical structure and potent biological abilities of tadehaginoside and HYD, their characteristic *in vivo* should be clarified. Up to now, the pharmacokinetics and tissue distribution of tadehaginoside in rats have never been carried out. Studying pharmacokinetics and tissue distribution can help to predict and clarify events related to drug efficacy and toxicity, and is an essential part of the drug development process (Li et al. 2015; Bhateria et al. 2016). Accordingly, the *in vivo* pharmacokinetics and tissue distribution of tadehaginoside in an applicable model and exploration of its properties would be extremely helpful.

Therefore, this work focussed on establishing a specific and reliable HPLC-MS/MS method so that it can be used in quantitatively determining the tadehaginoside and their metabolite in rats. This work analyzed the pharmacokinetic properties of tadehaginoside and their metabolite, which could provide a reference foundation for assessing the pharmacological activities of tadehaginoside and their metabolites.

## Materials and methods

### Reagents

The aerial parts of the *T. triquetrum* plant were collected from the Lingshui, Hainan province of China in July of 2017. The plant was identified by Professor Niankai Zeng (School of Pharmaceutical Science, Hainan Medical University, Hainan, China). Tadehaginoside (the purity is over 98%) was separated and purified from *T. triquetrum* by our research group. HYD (the purity is over 98%) was from Beijing Bailingwei Technology Co., Ltd. (Beijing, China). Quercetin, purchased from the National Institute for Food and Drug Control (Beijing, China), was used as the internal standard (IS, purity = 99.1%) for LC-MS/MS analysis. Ascorbic acid, CH_3_OH, and HCOOH were of HPLC grade and were obtained from Aladdin Reagents (Shanghai, China). Deionised water was sourced from Hangzhou Wahaha (Hangzhou, China).

### Instruments and LC-MS/MS conditions

The HPLC system was equipped with a SIL-20AC_XR_ autosampler, two LC-20AD_XR_ pumps, an online degasser, and a CTO-20A column oven, and they were all purchased from Shimadzu (Kyoto, Japan). The chromatographic column was Synergi™ Fusion-RP 80 Å C_18_ (4 μm, 2.10 mm i.d × 50 mm, Phenomenex, Torrance, CA, USA), the temperature was maintained at 40 °C during analysis. The aqueous solution containing 0.1% formic acid and methanol with 0.1% formic acid made up the mobile phase. The gradient elution was 10% B at 0–0.29 min, 90% B at 0.30–3.00 min, and 10% B at 3.01–4.00 min. The flow rate was set at 0.5 mL/min and the injection volume was 5 μL.

An AB Sciex Triple Quad™ 5500 system (Applied Biosystems, Foster City, CA, USA) was operated in the electrospray negative ionization mode (ESI^–^). To separate and determine tadehaginoside, HYD, and IS efficient, the MS analysis detection was optimized when the collision energy was at −22 V for tadehaginoside, −18 V for HYD and −29 V for IS, respectively. The optimized declustering potential was −120 V for tadehaginoside, 120 V for HYD, and −120 V for IS, respectively. Temperature, 550 °C; curtain gas, 25 psi; nebulizer gas, 55 psi; heater gas, 50 psi; ion spray voltage, −4500 V. The ion pairs of *m/z* 433.3→125.2 (tadehaginoside), *m/z* 162.8→119.0 (HYD) and *m/z* 301.1→151.0 (IS) were used for the quantitative analysis while undergoing multiple reactions monitoring (MRM).

### Animals

Animal studies were conducted with 180–240 g male Sprague-Dawley rats, which were purchased from Hunan Slack Jingda Experimental Animals (Hunan, China; approval number: SCXK (Xiang) 2016–0002). A total of 10 rats were randomly divided into two groups (5 animals in each group) for pharmacokinetic study. Group I received tadehaginoside by intragastric gavage at a single dose of 25 mg/kg. Group II received tadehaginoside by intravenous gavage at a single dose of 5 mg/kg. Twenty rats were randomly divided into five groups (4 animals in each group) for tissue distribution investigation. Tadehaginoside was given to the rats at a dosage of 5 mg/kg with intravenous administration. All rats were placed in cages in a room with a relative humidity of 50% and temperature at 23 ± 2 °C and were exposed to a 12 h light/dark cycle for a week before experiments. Animals fasted for 12 h before drug administration, and water could be obtained *ad libitum*. Experimental procedures on animals were undertaken following the National Guidelines and were approved by the animal ethics committee of Hainan Medical University (reg. no. 201506017/HMU).

### Preparation of IS and samples

#### Preparation of stock solutions and working solutions

A certain amount of IS, HYD and tadehaginoside was dissolved in methanol to obtain the stock solution at a concentration of 1 mg/mL. The working solution of IS was further handled by dilution with methanol of stock solution to a final concentration of 10 μg/mL. Next, a linear concentration gradient (1, 5, 10, 100, 1000, 5000, 10,000, and 20,000 ng/mL) of tadehaginoside stock working solution was serially diluted with methanol for pharmacokinetic studies and 50, 100, 1000, 5000, 10,000, and 20,000 ng/mL for tissue-distribution studies. Moreover, the concentrations of working solutions in plasma were 10, 50, 100, 500, 1000, 5000, 10,000, and 20,000 ng/mL for HYD. All solutions were kept refrigerated at 4 °C.

#### Preparation of calibration standards and quality control (QC) samples

Calibration standards and QC samples were prepared by mixing blank plasma or tissues with the working solutions. The concentrations of calibration standards ranged from 1–2000 ng/mL (1, 10, 100, 500, 1000, and 2000 ng/mL) for tadehaginoside in plasma, and 5–2000 ng/mL (5, 10, 100, 500, 1000, and 2000 ng/mL) in tissues. Similarly, a calibration curve was prepared in the range of 10 ng/mL to 2000 ng/mL at six concentration levels (10, 50, 100, 500, 1000, and 2000 ng/mL) for HYD in plasma. The final concentrations of QC samples were 3, 120, 1500 ng/mL for tadehaginoside in plasma samples, and 12, 120, 1500 ng/mL for tissue samples. In the same manner, three QC samples were set at 30, 120, 1500 ng/mL for HYD.

#### Preparation of sample solutions

The solution of ascorbic acid (5 μL) was transferred to rat plasma (50 μL) and then vortex-mixed for 15 s. Next, the sample was mixed with IS solution (5 μL, 10 μg/mL in methanol) and methanol (150 μL) and mixed for 1 min. After centrifugation (13,000 *g*, 10 min), 5 μL of the supernatant was injected into the apparatus.

To investigate its tissue distribution, each weighed tissue was homogenized in 0.9% NaCl (1:2, *w/v*) after thawing. Thereafter, 100 μL of the tissue homogenate and 10 μL of the ascorbic acid-saturated solution were added to a glass tube and mixed for 15 s. The IS working solution (5 μL, 10 μg/mL in methanol) and methanol (300 μL) were added to it in turn. After vortex-mixing for 1 min, then it was centrifuged at 13,000 *g* for 10 min under 4 °C. The subsequent steps were conducted according to the procedure applied above.

#### Method validation

The method in the present study was validated according to the FDA and other related guidelines (Chinese Pharmacopoeia Commission 2015; US Food and Drug Administration 2001). The rat plasma and target tissues were analyzed to assess the specificity. Plasma and blank homogenates of the livers and kidneys as representative samples were screened for linearity, precision, accuracy, recovery, matrix effect, and stability.

#### Specificity

Specificity was determined by testing blank rat plasma and tissue homogenates from different rats, tadehaginoside, HYD and IS mixed with biological samples, and biological samples collected after treatment with tadehaginoside, respectively.

#### Linearity and LLOQ

In the study, 5 μL of working solutions were added into 45 μL of blank rat plasma to prepare the standard plasma samples. After spiking different concentrations of working solution (10 μL) to blank tissue homogenates (90 μL), the tissue standard solutions were obtained.

Calibration curves were constructed according to the previous report. Briefly, the least-squares linear regression method with 1/*x*^2^ weighting was used to generate the slope, intercept, and correlation coefficient of each linear regression equation. The lowest concentrations (LLOQ) of tadehaginoside and HYD in the calibration curve were detected with an acceptable precision ≤ 20% and accuracy within ± 20%.

### Accuracy and precision

The accuracy and precision of the method in within-run and between-run conditions were evaluated using three consecutive batches and on more than two days at low, medium, and high QC levels (*n* = 6). The relative error (RE%) was applied to express the accuracy and the relative standard deviation (RSD%) was applied to express the precision.

#### Matrix effect

Blank plasma and tissues were processed, the QC samples were added, and the matrix effect in samples was analyzed in three levels (low, medium, and high). Next, the mean peak area of the analyte or IS in post-extracted spiked plasma/tissue homogenates was compared against the neat sample at the corresponding concentration.

The matrix factor (MF) of analytes (or IS) and IS-normalized MF were evaluated using Eqs. (1) and (2) (Bhateria et al. 2016; Ramakrishna et al. 2016).

(1) $$\mathrm{MF}\;=\;\frac{\text{Peak}\;\text{area}\;\text{of}\;\text{analyte}\;\text{in}\;\text{the}\;\text{presence}\;\text{of}\;\text{biomatrix}\;\text{components}}{\text{Peak}\;\text{area}\;\text{of}\;\text{analyte}\;\text{in}\;\text{the}\;\text{mobile}\;\text{phase}}$$

(2) $$\mathrm{IS}-\text{normalized MF }=\;\frac{\text{Peak}\;\text{area}\;\text{ratio}\;\text{of}\;\text{analyte}\;\text{to}\;\text{IS}\;\text{in}\;\text{the}\;\text{presence}\;\text{of}\;\text{biomatrix}\;\text{components}}{\text{Peak}\;\text{area}\;\text{ratio}\;\text{of}\;\text{analyte}\;\text{to}\;\text{IS}\;\text{in}\;\text{the}\;\text{absence}\;\text{of}\;\text{biomatrix}\;\text{components}}$$

#### Recovery

Recovery experiments were calculated *via* the determination of six replicates from the QC samples. The extraction recoveries were obtained by comparing the response of analytes from the extracted samples with the response of the same concentration of analytes spiked into the solution extracted from blank biological samples.

#### Stability

Stability was assayed by quintuplicate determinations of QC samples for each concentration. The following conditions were applied to test the stability of tadehaginoside and its metabolite: (i) after 4 h at room temperature (samples which had undergone a protein-precipitation procedure); (ii) after 2 h at room temperature (samples which had not undergone a protein-precipitation procedure); (iii) after 6 h in the autosampler (15 °C); (iv) after 24 h at 2–8 °C; (v) after three freeze-thaw cycles; (vi) after 7 days of storage at −20 °C.

#### Pharmacokinetic study

Two groups (*n* = 5 per group) were set up by randomly dividing the ten male Sprague-Dawley rats. One group was given tadehaginoside intravenously at a dose of 5 mg/kg. The other group was orally administered tadehaginoside at doses of 25 mg/kg. Blood samples (0.2 mL) were collected immediately from the suborbital vein and placed in heparinized 1.5 mL polythene tubes before drug administration (control group), 5.0, 10.0, 15.0, 20.0, 30.0, 45.0, 60.0, 90.0, 120.0, 240.0, and 360.0 min, respectively, after drug administration. Then, each blood sample was immediately centrifuged at 2000 *g* for 10 min at 4 °C, and plasma was harvested and stored at −20 °C until further treatment.

#### Tissue-distribution study

The tissue-distribution investigation was conducted on twenty Sprague–Dawley rats which were divided randomly into five groups. Rats were intravenous administration at a dose of 5 mg/kg and sacrificed by overdose of pentobarbital sodium (100 mg/kg) intraperitoneally for each time point (30.0, 60.0, 120.0, and 240.0 min). To remove superficial blood and contents, tissues (brain, heart, liver, spleen, lungs, kidneys, stomach, small intestine, skeletal muscle, body fat, and testes) were harvested and rinsed with ice-cold physiologic (0.9%) NaCl. Next, tissues were blotted with filter paper, weighed accurately, and homogenized in 0.9% NaCl (1:2, *m/v*). The obtained tissue homogenates were immediately stored at −20 °C until analysis.

#### Statistical analysis

To calculate pharmacokinetic parameters, DAS 3.2.8 (Mathematical Pharmacology Professional Committee of China, Shanghai, China) was applied as a non-compartmental model. The half-life, area under the curve, clearance rate and mean residual time were calculated. Results are all expressed as the mean ± standard deviation (SD).

## Results

### Method conditions

**Figure 1.**
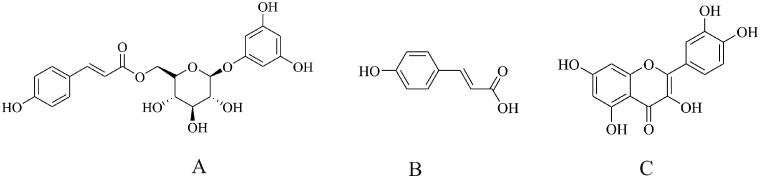
Structural formula of tadehaginoside (A), *p*-hydroxycinnamic acid (B) and quercetin (C).

An HPLC–MS/MS was established to investigate and optimize the separation of tadehaginoside, HYD, and IS. Chromatographic separation was conducted on a Synergi™ Fusion -RP 80 Å C18 column (4 μm, 2.10 mm i.d × 50 mm) with a mixture of the aqueous solution with formic acid (0.1%, *v/v*) and methanol containing formic acid (0.1%, *v/v*) as the mobile phase. Figure 1 depicts the chemical structures of tadehaginoside, HYD, and IS. The MRM with an electronic spray ionization source was performed to measure the response of tadehaginoside (*m/z* 433.3→125.2), HYD (*m/z* 162.8→119.0), and IS (*m/z* 301.1→151.0).

### Method validation

#### Specificity

**Figure 2.**
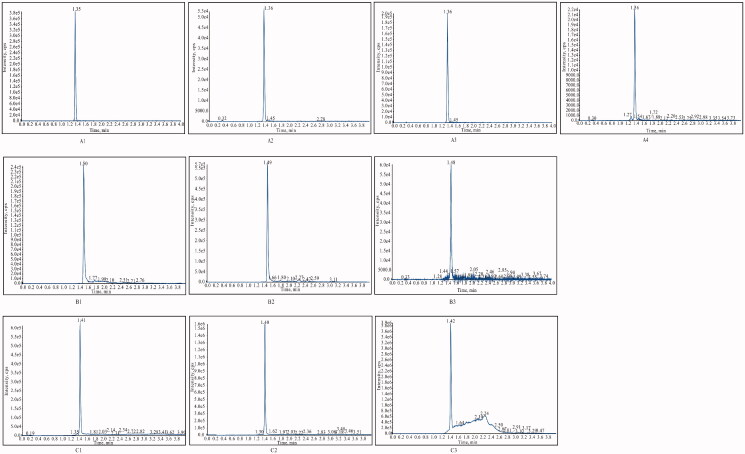
Typical chromatograms of different samples (A1) Tadehaginoside in blank plasma; (A2) Tadehaginoside in blank matrix (spleen); (A3) Plasma sample at 5 min after intravenous administration of tadehaginoside; (A4) Kidney sample at 30 min after intravenous administration of tadehaginoside; (B1) Blank plasma spiked with IS; (B2) Blank matrix (spleen) spiked with IS; (B3) Plasma sample at 5 min after intravenous administration spiked with tadehaginoside and IS; (C1) HYD in Blank plasma; (C2) Plasma sample at 5 min after intravenous administration spiked with HYD; (C3) Plasma sample at 5 min after intragastric administration 5 min spiked with HYD.

Typical MRM chromatograms of blank plasma and tissues, blank plasma and tissue homogenates spiked with analytes as well as real plasma and tissue homogenates samples after administration of tadehaginoside were shown in Figure 2. Endogenous interference was not detected at the retention time of tadehaginoside (1.35 min), HYD (1.41 min), and IS (1.50 min) owing to the high selectivity of the MRM mode.

#### Linearity and LLOQ

The linearity parameters for tadehaginoside and HYD are shown in Table 1. The correlation coefficient was over 0.99 for all of the calibration curves. The accuracy of the back-calculated concentrations in calibration curves for tadehaginoside was 87.7–121.9% in plasma, 87.8–109.5% in the kidneys and liver, and for HYD was ranged from 82.5%–119.5% in plasma. The RSD% was not more than 12.3% for tadehaginoside and HYD. The LLOQ of tadehaginoside was 1 ng/mL in plasma, 5 ng/mL in the kidneys and liver and for HYD was obtained as 10 ng/mL in plasma. These data suggested that our method was sensitive for both pharmacokinetic and tissue distribution studies.

**Table 1.** Parameters of standard curves of tadehaginoside and HYD in the rats as determined by LC-MS/MS during method validation.

Sample	Matrix	Run	Slope (10^–4^)	Intercept (10^–3^)	*r*
Tadehaginoside	Plasma	1	8.78	2.51	0.9967
		2	8.65	5.25	0.9985
		3	8.8	4.36	0.9984
	Liver	1	7.68	−1.22	0.9984
		2	7.37	7.03	0.997
		3	7.58	4.36	0.9988
HYD	Kidney	1	7.11	4.18	0.9985
		2	7.17	1.42	0.9974
		3	7.06	6.36	0.9962
	Plasma	1	16.1	1.69	0.9983
		2	17.1	2.85	0.9978
		3	17.9	2.99	0.9992

#### Accuracy and precision

Data regarding precision and accuracy are showed in Table 2. The within-run and between-run precision of tadehaginoside and HYD were less than 11.8% and 10.6%, respectively. Moreover, the within-run and the between-run accuracy of the method were determined lower than 109.2% and 107.2%.

**Table 2.** Accuracy, precision, matrix effect and recovery of the LC-MS/MS method to determined tadehaginoside and HYD in rat plasma and various tissues (*n* = 6).

Sample	Matrix	concentration (ng/mL)	Batch	Within-run		Between-run		Matrix effect		Recovery	
				Precision (RSD%)	Accuracy (% nominal)	Precision (RSD%)	Accuracy (% nominal)	Mean (%)	RSD (%)	Mean (%)	RSD (%)
Tadehaginoside	Plasma	1500	1	7.7	98	7.1	95.5	104.4	4.	79.9	7.8
			2	7.3	93.5						
			3	6.7	94.9						
		120	1	5.7	97.1	5.9	96.9	107.1	7.5	81.6	9.4
			2	6.9	97.3						
			3	6.1	96.3						
		3	1	8.2	96	9.1	98	108.4	10.6	80.2	4.3
			2	9.7	97.4						
			3	10.1	100.7						
	Liver	1500	1	7.4	97	8.8	99.8	95.3	1.2	116.0	3.9
			2	9.8	98.2						
			3	8.7	104.3						
		120	1	8.3	100.6	7.7	100.1	93.8	4.5	112.4	3.6
			2	8.7	98.2						
			3	7.2	101.5						
		12	1	9.5	109.2	8.5	107.2	104.7	7.4	104.8	8.6
			2	7.4	107.8						
			3	9.6	104.7						
	Kidney	1500	1	9.5	98.3	9.6	95.4	109.1	10.2	109.6	14.9
			2	9.1	91.6						
			3	10.3	96.5						
		120	1	6.4	98.2	6.6	98.2	100.8	13.1	106.7	5.6
			2	7.5	100.8						
			3	6	95.6						
		12	1	7.6	105.9	7.4	105.1	107.7	6.5	104.2	3.8
			2	8.6	103.5						
			3	7.3	105.7						
HYD	Plasma	1500	1	10.7	98.4	10.6	99.4	105.3	11.6	88.1	2.6
			2	11.0	100.6						
			3	11.8	99.1						
		120	1	7.4	98.6	8.1	98.94	103.4	5.7	84.6	9.6
			2	9.4	99.0						
			3	8.9	99.2						
		30	1	10.5	95.2	9.1	98.1	104.9	9.5	82.5	5.8
			2	9.0	98.9						
			3	8.7	100.1						

#### Matrix effects

Matrix effects were used to reflect the accuracy of the analysis results (Chamberlain et al. 2019). The mean IS-normalized MF was evaluated by using the same method in plasma and tissue homogenates with RSD% <15%, as shown in Table 2. No significant suppression or enhancement of ions due to matrix components was found, indicating the method of extraction met the requirements and the analytical method was reliable.

#### Recovery

In Table 2, the extraction recoveries of tadehaginoside and HYD at three working concentration levels in samples were found to be 80.2–116.0% and 82.5–88.1%, respectively. The results indicated that this simple sample preparation method for extraction recovery was high and stable.

#### Stability

Stability is one of the key parameters to be investigated during our study as it is meant to establish the conditions for storage and extraction. As shown in Table 3, the stability of analytes in different conditions was within acceptable levels. Tadehaginoside was stable in plasma and various tissues under the following conditions: after three freeze-thaw cycles; at room temperature for 2 h and 4 h; at 15 °C in the autosampler for 6 h; at 2–8 °C for 24 h (Recovery% ranged from 80.5% to 115.2% with RSD ≤ 9.4% for plasma, 81.2% to 110.4% with RSD ≤ 15.3% for tissue homogenates), and in a freezer set at −20 °C for 7 d (Recovery% ranged from 82.3% to 95.7% with RSD ≤ 11.5% for plasma, 87.7% to 99.8% with RSD ≤ 13.3% for tissue homogenates). For HYD, the plasma samples were stable with a Recovery% range of 83.3%–107.2% and RSD ≤ 13.9% at each storage condition.

**Table 3.** Stability of the tadehaginoside and HYD in rat plasma and various tissues under different storage conditions.

Sample	Matrix	Concentration (ng/mL)	Room temperature				Auto-sampler 6 h		Stored at 4 °C 24 h		Three freeze-thraw cycles		Storage at −20 °C 7 days	
			4 h		2h									
			Recovery (%)	RSD (%)	Recovery (%)	RSD (%)	Recovery (%)	RSD (%)	Recovery (%)	RSD (%)	Recovery (%)	RSD (%)	Recovery (%)	RSD (%)
Tadehaginoside	Plasma	1500	95.1	4.2	113.6	2.5	104.4	3.8	115.2	1.7	105.6	4.5	95.7	11.5
		120	107.0	9.4	99.2	6.9	96.1	6.6	100.8	9.1	97.2	6.5	94.4	5.4
		3	80.5	0.2	96.6	4.0	104.0	7.5	94.0	4.0	94.1	4.4	82.3	7.4
	Kidney	1500	101.5	4.8	106.0	4.6	105.3	7.3	103.1	6.4	103.6	7.1	96.6	5.7
		120	100.7	5.8	96.4	4.1	95.0	2.6	95.8	3.8	97.2	10.6	92.8	11.5
		12	85.7	9.9	81.2	10.3	89.8	12.7	87.8	5.5	92.0	10.2	87.7	13.3
	Liver	1500	102.4	5.2	104.9	4.4	104.3	9.4	110.4	5.2	103.4	4.3	99.8	3.9
		120	98.7	5.5	93.3	3.6	96.4	6.9	97.8	9.9	102.8	13.0	95.0	9.4
		12	89.9	14.2	88.7	15.3	90.7	3.2	89.1	11.0	90.2	10.0	88.6	3.6
HYD	Plasma	1500	106.0	1.9	102.2	5.3	104.4	7.8	107.2	6.4	102.5	3.9	107.2	2.7
		120	99.7	6.0	94.2	9.2	97.5	7.0	100.3	4.1	98.6	13.9	96.5	7.1
		30	86.0	6.2	85.6	6.0	84.1	1.5	84.6	5.1	83.3	4.0	80.9	2.6

#### Pharmacokinetic study and tissue-distribution study

**Figure 3.**
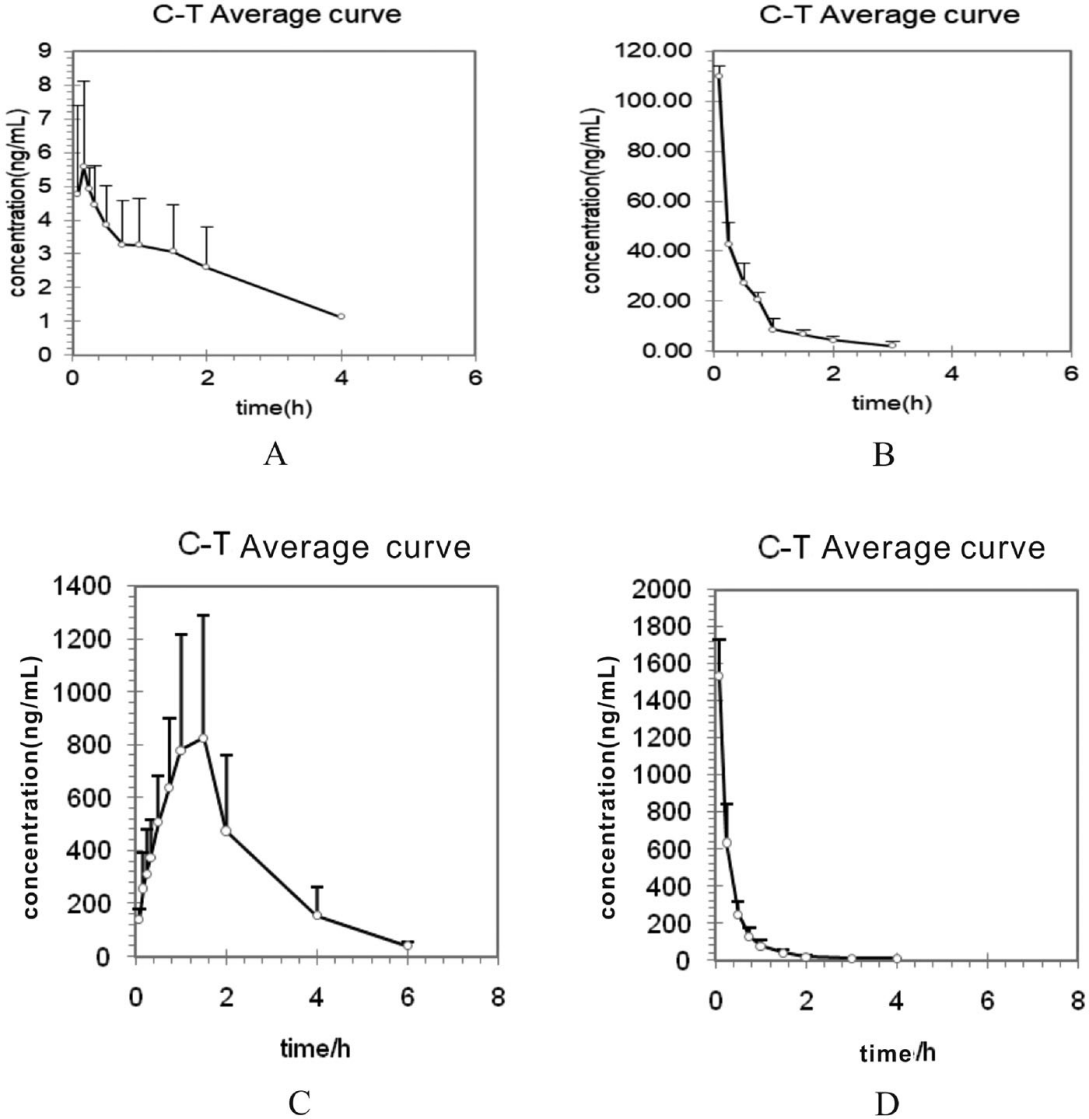
Mean plasma concentration–time curves of tadehaginoside and *p*-hydroxycinnamic acid after (A), (C) intragastric administration (25 mg/kg); (B), (D) intravenous administration (5 mg/kg) to rats.

**Figure 4.**
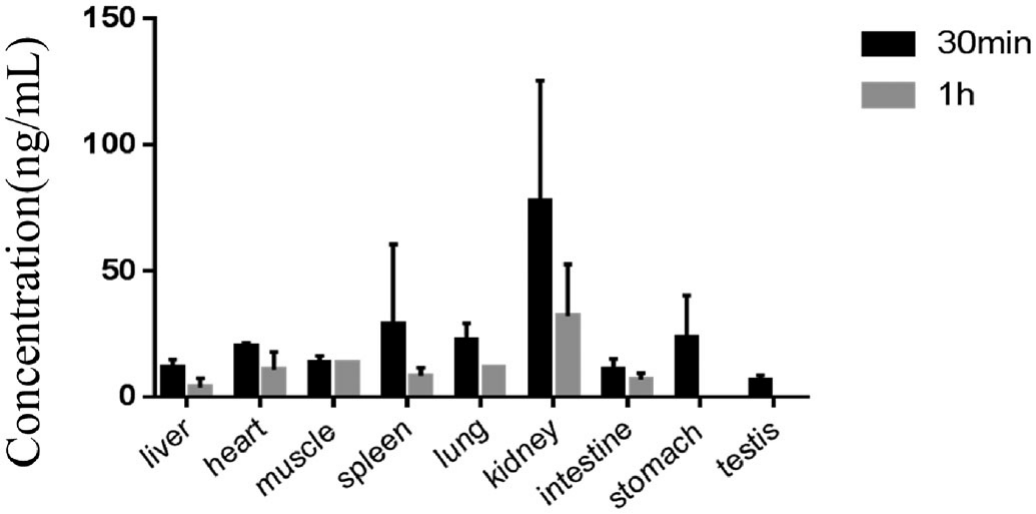
Concentration of tadehaginoside in rat tissues determined by HPLC-MS/MS.

The LC-MS/MS method was successfully used in investigating the pharmacokinetics of tadehaginoside and HYD, which followed a single dose (25 mg/kg for intragastric and 5 mg/kg for intravenous) administration. The major pharmacokinetic parameters of tadehaginoside and HYD were calculated by a non-compartmental model and demonstrated in Table 4. The mean plasma concentration-time curves as depicted in Figure 3. The concentrations of tadehaginoside in tissues determined at 0.5 and 1 h are shown in Figure 4.

**Table 4.** Pharmacokinetic parameters of tadehaginoside and HYD after intragastric and intravenous administrations.

PK parameters	i.g. (25 mg/kg)		i.v. (5 mg/kg)	
	Tadehaginoside	HYD	Tadehaginoside	HYD
*t*_1/2_ (h)	2.51 ± 2.21	1.24 ± 0.038	1.27 ± 1.19	0.86 ± 0.58
*C*_max_ (ng/mL)	6.01 ± 2.15	837.75 ± 446.66	109.77 ± 4.29	1536.45 ± 193.93
*T*_max_ (h)	0.25 ± 0.08	1.25 ± 0.43	0.08 ± 0.00	0.08 ± 0.00
AUC_0–_*_t_* (h ng/mL)	7.92 ± 5.43	2027.58 ± 1091.20	52.85 ± 5.11	597.24 ± 103.90
AUC_0–∞_ (h ng/mL)	14.12 ± 4.60	2099.87 ± 1103.33	59.22 ± 10.79	608.43 ± 98.42
MRT _(0–_*_t_*_)_ (h)	0.52 ± 0.44	1.84 ± 0.11	0.54 ± 0.07	0.39 ± 0.07
Vz (L/kg)	6089.77 ± 4011.02	30.63 ± 28.50	143.27 ± 102.37	10.96 ± 8.95
CLz (L/h/kg)	1937.91 ± 770.51	15.19 ± 9.74	86.44 ± 13.88	8.38 ± 1.28

## Discussion

### Optimization of method conditions

The stable-isotope labelled analogs of the analytes are usually used as IS. However, the isotope labelled analogs of tadehaginoside are challenging to synthesize and the cost is too expensive. In this study, different possible internal standards were tested including chlorogenic acid and quercetin as their chromatographic behaviours and extraction efficiencies were similar to those of tadehaginoside. Chlorogenic acid was found to be unsuitable owing to poor peak shape and strong interference in the MRM channels. To control the matrix effect, we used quercetin, which is believed to be the most appropriate IS for quantitative LC–MS/MS. Chromatography conditions such as the constitution of the mobile phase have a vital role in attaining a good result (for instance, appropriate ionization) (Millecam et al. 2019; Tao et al. 2019). In order to optimize the analytical performance, two different mobile phases which are methanol-water, acetonitrile-water are used. The result displayed that acetonitrile fell short of the target as the organic phase, but methanol could perform a perfect peak shape and a better resolution during the experiment. Moreover, the different concentrations of the organic reagent (50%, 60%, 70%, 80%, 90%, and 100%, respectively) that improved the speed of sample analyses and peak shape were investigated. The results showed that the mobile phase of water (containing 0.1% acetic acid)-methanol (containing 0.1% acetic acid) was a more suitable combination to achieve the optimal retention time and ionization of analytes.

**Figure 5.**
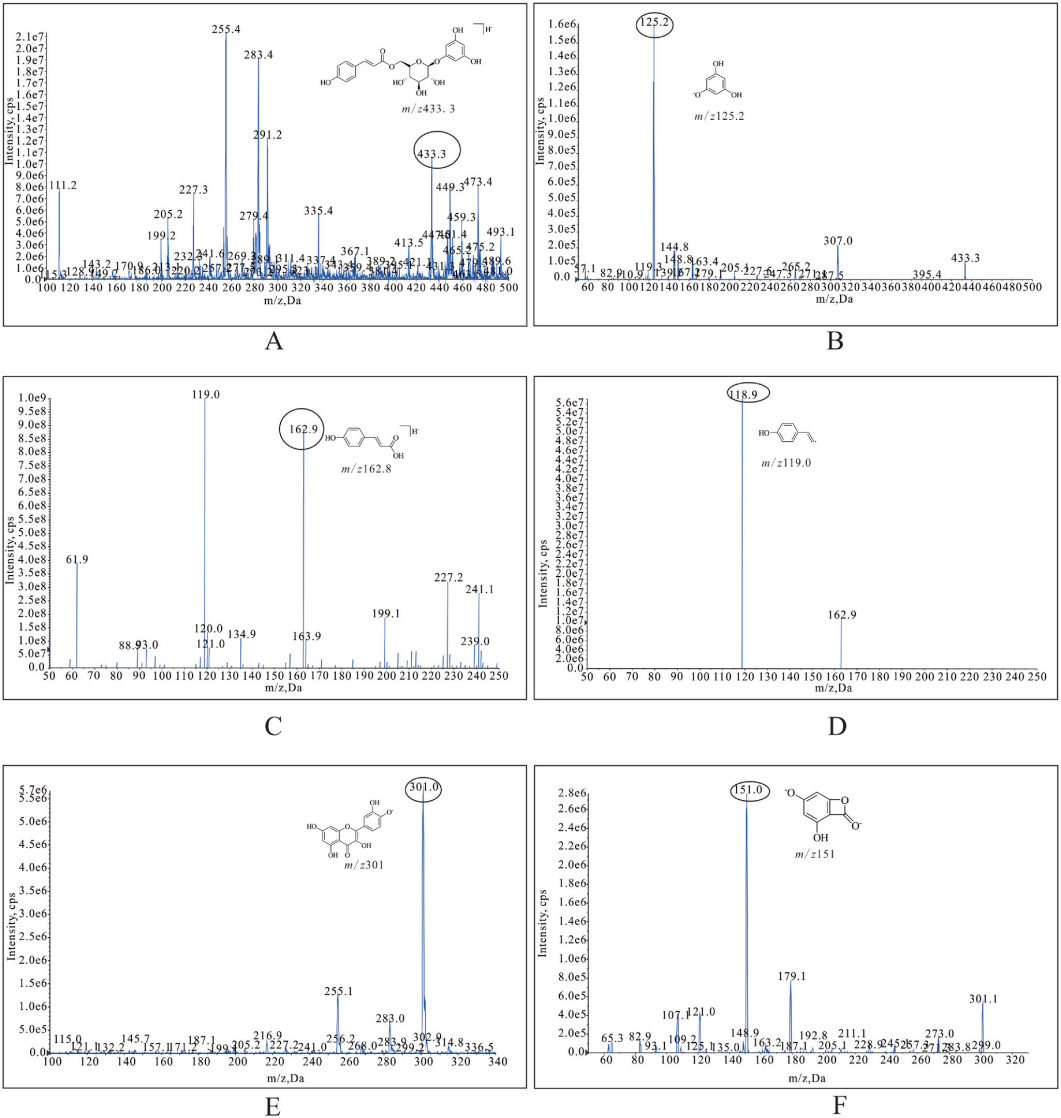
The mass spectrum of tadehaginoside [(A), Q1-scan; (B) Full-scan], *p*-hydroxycinnamic acid [(C), Q1-scan; (D) Full-scan] and quercetin [(E), Q1-scan; (F) Full-scan].

The MS conditions were optimized to achieve high recovery, sensitivity, and selectivity. The mass spectrum of tadehaginoside, HYD and IS are displayed in Figure 5. In Q1 scan mode, singly charged protonated precursor ion [M-H]^-^ of tadehaginoside was found to be *m/z* 433.3. In product ion scan, abundant product ion was observed at *m/z* 125.0 or 125.2 for tadehaginoside. Therefore, the MRM transition of *m/z* 433.3→125.0 was used for the quantification of tadehaginoside. Next, the transition ion of *m/z* 162.9→119.0 for HYD was determined in the same way. Finally, the MS conditions of quercetin initially were based on the literature for IS (Day et al. 2001). In order to obtain the highest relative abundance of precursor and product ions from the MS/MS product ions of analytes, the parameters such as fragment energy and collision energy were optimized, respectively.

### Sample preparation

In the selection of treatment conditions for plasma samples, the peaks of tadehaginoside and quercetin of those samples without an ascorbic acid solution in the protein precipitation approach were degraded to different degrees after being placed at room temperature or in the automatic sampler for a long time. This phenomenon was especially shown in quercetin. Therefore, a suitable amount of ascorbic acid solution was added during plasma protein treatment to make the sample more stable.

### Pharmacokinetic study

Following intragastric administration at a dose of 5 mg/kg, the peak drug concentration (*C*_max_) of tadehaginoside was 6.01 ± 2.14 ng/mL. Comparing with intravenous injection, the *C*_max_ of tadehaginoside showed a relatively low concentration after intragastric administration, suggesting that the absorption of tadehaginoside was not good in the gastrointestinal tract. The result was similar to the absorption behaviour from most glycosides (Azuma et al. 2000; Liu et al. 2009). Tadehaginoside, as well as most glycosides, consists of multiple hydrophilic groups (like hydroxyl groups) in their molecular structure, which leads to hydrophilicity greater than lipophilicity. Therefore, it was difficult to pass through the biofilm and has a low enrolment concentration. At 0.08 h (*T*_max_) after intravenous administration, *C*_max_ was found to be 109.77 ± 4.29 ng/mL, indicating the quick absorption of tadehaginoside in rats in this way. We also found that tadehaginoside was 5 ng/mL, while the concentration of HYD achieved a high level for intragastric administration at 0.08 h. As the existence of multiple hydrolyzes in plasma, such as carboxylesterase, amide ester hydrolase, phosphatase, epoxide hydrolase and so on, we speculated that tadehaginoside could be hydrolyzed rapidly after intravenous administration resulting in low concentration. The results showed that HYD is an active metabolite *in vivo* after oral tadehaginoside and the pharmacological activities of tadehaginoside such as antidiabetic and antihyperlipidemic might be derived from HYD. And the *t*_1/2_ of tadehaginoside was 2.51 ± 2.21 h for intragastric and 1.27 ± 1.19 h for intravenous administration, respectively. It was obvious that the blood concentration can be maintained for a long time after intragastric administration. The phenomenon suggests that tadehaginoside could be absorbed not only in the stomach but also in the small intestine, colon and other intestinal sites. There was no significant difference in the mean residence time from the time of dosing to the time of the last measurable concentration (MRT_(0−_*_t_*_)_) between intragastric and intravenous administration, which showed that tadehaginoside has better distribution and moderate elimination *in vivo*. A pharmacokinetic study indicated that tadehaginoside in plasma of rats with different administrations showed a certain difference in the pharmacokinetic parameters. In order to take better advantage of tadehaginoside, the forms of administration and the levels of dose still need to be validated to improve patients' compliance by further experiments. The bioavailability of tadehaginoside was evaluated according to these data which guided its further clinical research.

### Tissue-distribution study

The pharmacology of some drugs is different may be due to gender differences (Jakutiene et al. 2007; Liu et al. 2018), male SD rats were only selected in our study as they have relatively stable physiological characteristics and are more conducive to follow-up studies.

After intravenous administration of 5 mg/kg, we measured the concentrations of tadehaginoside in tissues at the designated time points of 0.5, 1, 2, and 4 h. Since the presence of tadehaginoside was detected in various tissues, we concluded that tadehaginoside can be widely distributed in rat tissues after intravenous administration, and these data were consistent with those of the pharmacokinetic study. At a dose of 5 mg/kg, the highest level of tadehaginoside was observed in all tissues except the brain, which revealed that tadehaginoside was difficult to cross the blood-brain barrier. Thirty minutes after intravenous administration, the observed distribution of concentration was (in descending order) kidneys > spleen > lungs > heart > muscle > liver > intestine. The higher levels in the kidneys and spleen could be due to the higher blood flow in these organs. Tadehaginoside was detected in the testes, stomach, and body fat of only individual rats might be explained by the individual differences in animals. At 2 h after dosing, the tadehaginoside concentration in tissues or organs was lower than the LLOQ. This result indicated that tadehaginoside was distributed rapidly and there was no long-term accumulation in most tissues, which was congruent with the observed change trend in the plasma concentration. Revealing the accumulation of tadehaginoside in the body by comparing the distribution of tadehaginoside in organs or different tissues at different time points could provide a reference for evaluating the target organs of tadehaginoside.

## Conclusion

To our knowledge, we present the first comprehensive pharmacokinetics and tissue-distributions study of tadehaginoside in rats after intragastric and intravenous administration. A specific, sensitive, and reliable LC-MS/MS method was established to determine tadehaginoside and their metabolite in the plasma and tissues of rats. Our method has significant advantages in terms of simple preparation of samples and short analysis times. We conclude that tadehaginoside could be distributed widely and eliminated rapidly after administration. One of the important targets in our next research could relate to taking some reasonable and effective methods to improve the oral bioavailability of tadehaginoside. The present study will contribute tadehaginoside to be a promising natural product, which also provides insights into further pharmacological investigations.

## Author contributions

ZHS and XPZ conceived and organized the review. CYZ, YTL and NM performed the experiments and drafted the manuscript. YFT, DL, QBL and WYL revised the manuscript. All authors read and approved the final manuscript.
